# Do Simultaneously Viewed Objects Influence Scene Recognition Individually or as Groups? Two Perceptual Studies

**DOI:** 10.1371/journal.pone.0102819

**Published:** 2014-08-13

**Authors:** Christopher R. Gagne, Sean P. MacEvoy

**Affiliations:** Department of Psychology, Boston College, Chestnut Hill, Massachusetts, United States of America; National Institute of Mental Health, United States of America

## Abstract

The ability to quickly categorize visual scenes is critical to daily life, allowing us to identify our whereabouts and to navigate from one place to another. Rapid scene categorization relies heavily on the kinds of objects scenes contain; for instance, studies have shown that recognition is less accurate for scenes to which incongruent objects have been added, an effect usually interpreted as evidence of objects' general capacity to activate semantic networks for scene categories they are statistically associated with. Essentially all real-world scenes contain multiple objects, however, and it is unclear whether scene recognition draws on the scene associations of *individual* objects or of object *groups*. To test the hypothesis that scene recognition is steered, at least in part, by associations between object groups and scene categories, we asked observers to categorize briefly-viewed scenes appearing with object pairs that were semantically consistent or inconsistent with the scenes. In line with previous results, scenes were less accurately recognized when viewed with inconsistent versus consistent pairs. To understand whether this reflected individual or group-level object associations, we compared the impact of pairs composed of mutually related versus unrelated objects; i.e., pairs, which, as groups, had clear associations to particular scene categories versus those that did not. Although related and unrelated object pairs equally reduced scene recognition accuracy, unrelated pairs were consistently less capable of drawing erroneous scene judgments towards scene categories associated with their individual objects. This suggests that scene judgments were influenced by the scene associations of object groups, beyond the influence of individual objects. More generally, the fact that unrelated objects were as capable of degrading categorization accuracy as related objects, while less capable of generating specific alternative judgments, indicates that the process by which objects *interfere* with scene recognition is separate from the one through which they *inform* it.

## Introduction

It has often been demonstrated that the context in which objects are viewed can strongly influence how accurately they are recognized. Objects are more readily recognized when viewed in the context of other objects with which they commonly co-occur, and when embedded in scenes of the category they are typically found in [1]–[14]. For example, a briefly presented fire hydrant is more readily recognized when presented against an urban streetscape background than when placed in a pastoral setting.

At the same time, it is intuitive that judgments of scenes' own categories are influenced by the kinds of objects they contain. To a certain extent this must intuition must be correct, since some types of scenes are essentially defined on the basis of their containing a single object type: a bedroom is a bedroom because it contains a bed, for instance. Even beyond such definitional situations, however, the statistical regularities of visual environments means that even those objects that do not *define* a scene category may still be *informative* of a scene's identity [15]. For instance, although consumer-grade coffee makers may be found in many different types of scenes, including offices and waiting rooms, they are most often encountered in kitchens, and it is therefore more likely that a room containing one is a kitchen than a member of any other category, all other factors being equal. It is therefore not surprising that the removal of informative objects from scenes can hamper scene categorization [16], as can the accidental presence or intentional insertion of incongruent objects [8], [9], [17]–[19].

Considerable theoretical and experimental effort has been devoted to understanding how recognition of objects is sculpted by their scene context [20]–[22]. Although the mechanism by which objects influence scene recognition has received somewhat less attention, it has generally been attributed to the activation by salient objects of schemata or context frames of scene categories they are statistically associated with [8], [20]. One complicating factor, however, is the fact that essentially all real-world scenes contain multiple objects, each one of which is theoretically capable of activating the context frame of the scene most closely associated with it. Previous research demonstrates that the insertion into scenes of pairs of objects that are either both consistent or inconsistent with the scene has no greater effect on scene categorization accuracy than insertion of a single consistent or inconsistent object [9], suggesting that multiple objects are ultimately “distilled” into unified scene schemata. But how does this unification take place?

One straightforward possibility is that each object in a multi-object scene independently triggers the context frame of its associated scene, if any. (Whether the proximal triggers are extracted labels for objects' semantic identities or simply their visual features is not important for present purposes.) From the resulting pool of activated schemata a “winner” is identified according to some set of criteria (e.g., the context frame receiving the most single object “votes”, the one with the greatest peak association across the object pool, etc.), perhaps applied as a part of an iterative winnowing process. For scenes in which all objects are associated with the same scene, this process terminates quickly; in the somewhat more interesting case of scenes containing objects with conflicting associations, it may take somewhat longer. Regardless of the exact selection criteria involved, a core characteristic of any such mechanism is that it will inevitably produce an active scene schema so long as one of the objects possesses a statistical scene association exceeding some threshold. Because schemata are initially triggered and subsequently selected on the basis of their associations with *individual* objects, we shall refer to this as *item-level* schemata triggering.

A potential alternative to this process is one in which context frames are not triggered by the statistical scene associations of individual objects, but by the statistical associations of object *groups*. For example, a judgment of “office” for a scene containing a computer and a coffee maker might arise not from some operations on the scene schemata the objects individually activate (“office” and “kitchen”, respectively), but from the activation of the single schema most closely associated with the “computer and coffee maker” combination. We shall refer to this process as *group-level* schemata triggering.

At first, the distinction we draw between item- and group-level schema activation may appear to be one without a substantive difference, inasmuch as both produce a single activated schema whose identity is dependent on the suite of objects visible in a scene. Indeed, under most circumstances we expect the two processes to produce the same outcome; in the trivial case of a scene containing a single object, they are logically identical. There is a critical difference, however. As already stated, the item-level model will always result in an active schema as long as a scene contains at least one object possessing a suprathreshold statistical association with a scene category (or, more properly, a peak of suprathreshold prominence in its profile of associations across all scene categories). In contrast, the group-level model leaves open the possibility that some groups of objects may fail to trigger any schemata at all, even when individual objects within them are strongly associated with one scene category or another. This might be expected to occur when, for instance, a scene contains two objects that appear together so rarely as to negate the possibility that either of their individual scene associations has any validity. In other words, the group-level model takes into account not just the statistical relationships between objects and scenes when activating schemata, but also the statistical relationships of the objects to each other to discern whether *any* schema is even warranted.

The ability of group-level triggering to return a “null schema” is potentially useful. Consider, for instance, the problem of discerning the category identity of an unfamiliar room being used by a home's occupants for storage, and hence containing an odd assemblage of objects that includes a large filing cabinet and a spare refrigerator. Let us assume that these two objects are situated near enough to each other that both are captured by an observer's initial glance into the room. Although these objects might individually be strongly associated with the categories of “office” and “kitchen”, respectively, the very strength of these disparate associations means that the two are rarely, if ever, observed together and therefore that a room containing both of them is relatively unlikely to belong to either category. Under the item-level model, schemata for each scene category are nonetheless activated and winnowed down to a single survivor, the presence of which potentially impedes arrival at the final judgment that the rooms is, in fact, neither a kitchen nor an office. In contrast, under the group-level model the absence of any scene associated with the filing cabinet/refrigerator pair can be expected to result in the generation of no object-based schema at all. This graceful failure means that the results of any ensuing detailed search of the room's contents will not have to compete with the strong individual scene associations of the salient objects, enhancing both the speed and accuracy of the scene's recognition relative to the item-level model. Because non-object features of scenes, such as global properties like texture and spatial layout, provide information about scene category that may in fact be temporally prioritized over objects during scene recognition [23]–[29] and may even sculpt perception of the objects themselves, we acknowledge that the benefit arising from the availability of a null schema may be minimal if one of the objects in a scene is clearly semantically incompatible with the scene's global properties. However, in situations where global properties provide comparatively little information about scene identity, as is often the case when distinguishing between categories of indoor rooms [30], the capacity of group-level triggering to use object-object statistical associations to avoid activation of implausible scene schemata is likely valuable.

The goal of the present study was to seek evidence that scene schemata are activated, at least in part, by objects evaluated as groups. In an experimental design adapted from two previous studies of the influence of objects on scene recognition [8], [9], observers viewed briefly-presented pairs of objects followed by complete scenes, whose identities observers were asked to determine. Previous work has demonstrated that recognition of scenes accompanied by pairs of objects that are inconsistent with the scenes but *episodically related to each other* (i.e., associated with the same scene) suffers relative to that of scenes accompanied by pairs composed of objects that are both consistent with the scenes they accompany [9]. In the present study, we extended this research by examining the impact on recognition of pairs consisting of objects that were inconsistent with the accompanying scene *and also unrelated to each other*. These were pairs which, evaluated as groups, were only weakly associated with any particular scene category, even though their constituent objects, evaluated as individuals, had strong scene associations. Based on this dissociation, we predicted that any degree of group-level triggering of scene schemata would be evident as a significantly smaller impact on scene recognition by inconsistent pairs composed of unrelated objects versus pairs composed of related objects. As reported below, we obtained conflicting results: while related and unrelated pairs produced similar numbers of errors, consistent with item-level triggering, they produced differing patterns of errors, consistent with group-level triggering. We reconcile this conflict by proposing that the effect of objects on scene recognition is brought about by separate disruptive and constructive processes that differ in their balance of item- versus group-level processing of objects.

## Methods

### Ethics Statement

This research was approved by the Institutional Review Board of Boston College, and conducted in accordance with the Declaration of Helsinki. Participants provided written informed consent and received course credit as compensation.

### Experiment 1

#### Participants

Participants were 20 Boston College undergraduate students, all with normal or corrected-to-normal vision.

#### Stimuli

Scene stimuli were 48 color photographs (640×480 pixel resolution) of easily identified scene categories, collected from the internet; there was one image per scene category. Objects were 96 color photographs of decontextualized objects, composed of two objects associated with each scene category. Scene categories and their associated objects are listed in Table 1. Grid-scrambled versions of all object and scene images were generated to serve as masks. Participants' heads were positioned by a chinrest such that scenes subtended 17.2°×13°. Each object was scaled isotropically such that its longer cardinal axis subtended 6.75°. Objects were shown in pairs (see below for details) in which each object was centered 5.4° to the left or right of the vertical meridian; object masks were centered on the same positions.

**Table 1:** Scene and associated object categories used in Experiment 1.

*Scene*	*Object 1*	*Object 2*	*Scene*	*Object 1*	*Object 2*
**casino**	poker chips	cards	**ice rink**	skates	hockey stick
**track**	hurdle	starting blocks	**restaurant**	Champaign bucket	salad
**warehouse**	ladder	forklift	**train station**	newspaper stand	clock
**bar**	pool table	dart board	**attic**	cardboard box	antique table
**construction site**	hard hat	excavator	**kitchen**	microwave	blender
**pool**	chair	swimsuit	**patio**	table with chairs	grill
**beach**	umbrella	ball	**bathroom**	laundry basket	scale
**gym**	bike	bench	**cafeteria**	stack of chairs	vending machine
**supermarket**	shopping cart	grocery bag	**workshop**	saw	level
**church**	priest	bible	**backyard**	rake	lawn mower
**playground**	bench	dog on leash	**basketball court**	water bottle	basketball
**ship**	coiled rope	barrel	**library**	magazine rack	books
**locker room**	cleats	towels	**football field**	football	goal post
**office**	filing cabinet	copier	**garden**	hose	watering can
**ski resort**	skis	gondola lift	**tennis court**	tennis racket	tube of tennis balls
**classroom**	globe	backpack	**farm**	tractor	cow
**movie theater**	popcorn	tickets	**garage**	jumper cables	toolkit
**skate park**	scooter	skateboard	**airport**	suitcase	baggage carriage
**baseball diamond**	baseball bat	pitcher	**desert**	camel	scorpion
**living room**	vacuum cleaner	television	**factory**	push cart	crate
**stream**	goose	canoe	**hospital**	wheel chair	blood pressure cuff
**parking lot**	trash can	truck	**polar**	penguin	polar bear
**porch**	potted plant	rocking chair	**golf course**	golf clubs	golf cart
**stage**	cello	grand piano	**jungle**	parrot	monkey

#### Procedure

**Figure 1:**
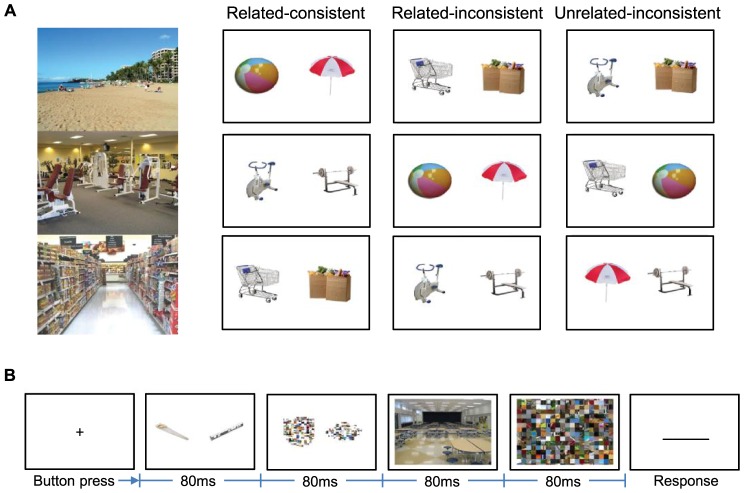
Visual stimuli and procedure. (a) Examples of scenes from categories of (top to bottom) beach, gym, and supermarket, and object pairs of the three types for each scene. (b) Temporal sequence of stimuli in each trial in Experiment 1. Trials were self-initiated by participants after acquisition of a central fixation point. Responses were entered by keyboard.

Each trial consisted of a pair of objects and a scene viewed in rapid succession. Objects in each pair could be 1) related to each other and consistent with the scene (referred to hereafter as *related-consistent* pairs), 2) related to each other but inconsistent with the scene (*related-inconsistent*) or 3) unrelated to each other and inconsistent with the scene (*unrelated-inconsistent*) (Figure 1A). No “unrelated-consistent” condition was used because one cannot logically be constructed; two objects that are unrelated to each other cannot, by our definition of relatedness, both be consistent with the same scene category. Similarly, we did not use any pair in which one object was consistent with the scene but the other was inconsistent, since this would have left the relationship between the pair and the scene indeterminate.

Each trial was initiated by the participant with a button press made while s/he fixated a central white cross. A pair of objects was then shown for 80 ms, followed by a pair of object masks for 80 ms, a scene for 80 ms, and a scene mask for 80 ms (Figure 1B). Immediately after the scene mask, a text box appeared on the screen into which participants were instructed to type the identity of the scene as quickly as possible. This procedure closely follows the design of Davenport [9], with two notable exceptions. First, we used unrelated-inconsistent pairs in addition to related-consistent and related-inconsistent. Second, whereas Davenport [9] embedded objects in scenes, in the present experiment they were shown immediately before scenes. This asynchrony was used to emphasize the relationship between objects in pairs by temporally severing relationships between object in pairs and scenes' endogenous objects.

Each participant performed 48 trials in each of 2 blocks, within which there were an equal number of related-consistent, unrelated-consistent, unrelated-inconsistent, and “mask” trials in which object pairs were replaced by pairs of object masks. Each of the 48 scene exemplars and 96 object exemplars was shown exactly once in each block, and therefore appeared in only one of the four condition types. The type of condition that each object and scene appeared in was the same in both blocks, but trials in each block followed a different random sequence. Objects and scenes were assigned to each condition type according to a series of three-way groupings of scene categories. Figure 1A contains an example of one such grouping, composed of the scene categories “beach”, “gym”, and “supermarket”. To form related-inconsistent trials, a scene was selected from each group and the two objects associated with one of the other scenes in the group was assigned to it; to form unrelated-inconsistent trials, a scene was selected and one object from each of the other two scene categories in the group were assigned to it. The three-way scene groupings were adopted as a tractable solution to the problem of ensuring that each object was shown exactly once in each block. However, because some objects could be considered somewhat consistent with more than one scene (e.g., a grocery bag might be considered consistent with both a supermarket and a kitchen), it was not practical to generate new random groupings for each participant. Instead, groupings were optimized to make certain that all objects were either distinctly related or unrelated with scenes they could appear with, and kept fixed across participants. Any bias introduced by these fixed groupings was tempered by the fact that only 25% of participants viewed any particular object/scene pairing. That is, although a photo of a gym was always preceded by a pair consisting of a beach ball and a shopping cart whenever it was selected to be shown as a part of an unrelated-inconsistent trial, it was only selected to serve in that role for 25% of participants; the remaining participants saw the gym as a part of a related-consistent trial, as a part of a related-inconsistent trial, or as a part of a mask trial, in equal proportions. When appearing in an unrelated-inconsistent trial, each scene was only ever shown with one of the four possible unrelated object pairs. For instance, all participants who saw the image of a gym as a part of an unrelated-inconsistent trial only saw it accompanied by the beach ball/shopping cart object pair (i.e., objects from the beach and supermarket categories), and never with the logically eligible umbrella/grocery bag, etc., pairs. Similarly, scenes in related-inconsistent trials only ever appeared with objects from one of the other two scene categories in each three-way grouping. These restrictions were necessary to avoid some scene/object combinations whose consistency was potentially ambiguous.

To determine scene recognition accuracy, scene responses entered by each participant were manually compared to a list of category names generated by that participant in a naming session that came at the end of his or her experiment session, in which s/he was allowed to view each scene by itself for an unlimited time before naming it. Scoring was blind to trial type.

### Experiment 2

As detailed in the Results, Experiment 1 revealed potentially informative differences in the types of errors produced by unrelated- and related-inconsistent trials, specifically the relative proportions of erroneous category judgments that matched the scene category associated with objects in each pair. Experiment 2 was designed to boost the overall rate at which this particular type of error was committed by limiting participant's response options. This was accomplished by reducing the stimulus set to include only four categories of scenes, and switching to a forced-choice task.

#### Participants

Participants were 18 Boston College undergraduate students, all with normal or corrected-to-normal vision.

#### Stimuli

Scene stimuli were 20 exemplars each of kitchens, bathrooms, offices and gyms. These categories were all used in Experiment 1. Object stimuli were images of 24 different objects associated with each of the four scene categories (Table 2). This expansion of the object set relative to Experiment 1 was due to our inability to acquire, for each of the two objects associated with each scene category in Experiment 1, 12 exemplars that we felt were of sufficient quality for the experiment. However, a comparison of Tables 1 and 2 reveals no obvious systematic differences in the strength of scene associations of the objects used in Experiments 1 and 2. All images were shown in grayscale to avoid providing global color cues to scene identity; this was not a concern in Experiment 1 owing to the greater number of scene categories used, and the single repetition of each.

**Table 2:** Objects associated with each scene category in Experiment 2.

*Bathroom*	*Office*	*Kitchen*	*Gym*
toilet bowl cleaner	copy machine	tea pot	machine press
shampoo bottle	stapler	wooden spoon	exercise bike
scale	desktop computer	stove	combination machine
sink	folder	refrigerator	elliptical machine
toilet	highlighter	dishwasher	treadmill
bathtub	briefcase	plate	gym bag
shower head	filing cabinet	fork	exercise bands
shower rack	hole punch	bowl	row machine
toothbrush	printer	microwave	running shoes
hair dryer	paper shredder	blender	medicine ball
hand soap bottle	pencil sharpener	cutting board	dumbbells
towel rack	pen	knife block	incline bench
rubber duck	sticky notes	rolling pin	abs machine
laundry basket	desk trashcan	frying pan	bench
bath mat	water cooler	pot	gym towel
lotion bottle	desk chair	toaster	barbell
hair brush	projector	baking tray	weight rack
hair straightener	office phone	wine bottle	lifting gloves
tissue box	calculator	spatula	abs roller
toilet paper roll	tape	measuring cup	head band
loofah	day planner	tea cup	medicine ball rack
soap bar	desktop lamp	spice rack	Olympic barbell
floss container	paper ream	water pitcher	yoga mat
folded towels	coffee travel mug	oven mitt	stop watch

#### Procedure

Stimulus trials contained the same sequence of events as in Experiment 1, except that scenes were shown for only 64 ms, which pilot studies showed produced a significant reduction in accuracy relative to the 80 ms presentation time used in Experiment 1. In addition, the presentation time for object pairs was shortened to 32 ms to reduce the possibility that participants could complete the task by ignoring scenes and paying attention solely to objects. Presentation times for masks were the same as in Experiment 1. Finally, the open response used in Experiment 1 was replaced by a four-alternative forced-choice. Participants were presented with a list of the four scene categories, labeled A-D, from which they selected their judgments. The order of names was randomized for each trial. Using the same nomenclature as Experiment 1, all trials were related-consistent, related-inconsistent, or unrelated-inconsistent. There was no “mask” trial type. Each participant performed a total of 200 judgments across all trial types.

#### Statistical analyses

Because they were attached to distinct hypotheses, all t-tests presented for both experiments were one-tailed unless otherwise noted.

## Results

### Experiment 1

**Figure 2:**
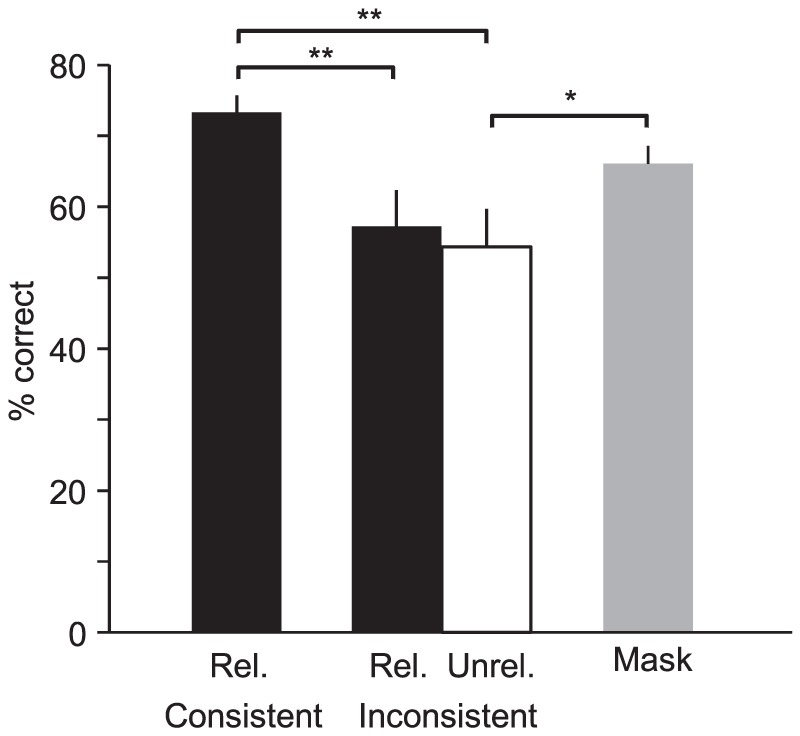
Results of Experiment 1. “Rel.” and “Unrel.” denote related and unrelated object pairs, respectively. Error bars are s.e.m. All pairwise significant differences between means are shown. *, p<0.05; **, p<0.01.

Average scene recognition accuracies for each condition type are presented in Figure 2. A repeated-measures ANOVA revealed a significant effect of condition type (F(3,57) = 8.75, p = 0.00007). Post-hoc tests showed that scene recognition accuracy was significantly lower in related-inconsistent trials than in related-consistent trials (Tukey HSD, p<0.01), replicating previous results obtained with inconsistent objects that were embedded in scenes [8], [9].

The lower accuracy on related-inconsistent trials is consistent with the general idea that the associations of objects with particular scene categories activated scene schemata that interfered with recognition of the subsequent actual scenes. It does not tell us, however, whether the interfering schemata were activated by objects evaluated as individuals or jointly as groups. To address this, we compared accuracy on related-inconsistent trials to accuracy on unrelated-inconsistent trials. Individual objects in these two trial types were equivalently associated with their respective scene categories, but *pairs* in related-consistent trials were clearly associated with a scene category while pairs in unrelated-inconsistent trials were not. Thus if the degraded scene recognition accuracy we observed with related-inconsistent pairs stemmed, at least in part, from schemata triggered by object groups, we expected accuracy to be less degraded by unrelated-inconsistent pairs.

This prediction was not borne out: recognition accuracy with unrelated-inconsistent objects was actually numerically lower than accuracy with related-inconsistent objects, although this difference was not statistically significant (Tukey HSD, p>0.05). This suggests that it was only the associations of individual objects with scene categories that controlled pairs' capacity to interfere with recognition of subsequent scenes. Thus, the comparison of *total* error rates provides no evidence for group-level triggering of scene schemata.

A potentially different picture emerged, however, when we examined the *patterns* of errors produced by inconsistent pairs. Both related- and unrelated-inconsistent trials generated above-chance numbers of “intrusions”, erroneous scene judgments that matched the category associated with one (for unrelated-inconsistent trials) or both (for related-consistent trials) of the objects in a pair [8], [9]. Consistent with previous studies, intrusions were relatively rare, with participants committing an average of 2.6 intrusions over the combined 48 trials that involved inconsistent pairs. However, intrusions accounted for 28.3% of total errors committed on these trials, far exceeding that expected from random guessing, even after making the generous and unsupportable assumption that participants somehow limited their guesses to the 48 scene categories tested (chance intrusion rate for related objects = 1/47, or 2.1%; chance rate for unrelated objects = 2/47, 4.2%).

What is particularly noteworthy, however, is that intrusions were not equally distributed between related- and unrelated-consistent trials. While the small number of intrusions committed by individual participants made a group-level comparison of intrusion rates difficult (13 of 20 participants committed either one or zero intrusions), after pooling errors across participants we found that intrusions accounted for 30 of 81 errors committed on related-inconsistent trials (37.0%), but only 21 of 99 errors committed on unrelated-inconsistent trials (21.2%). This difference in frequencies was significant (*χ* ^2^(1,180) = 5.49, p = 0.019; p-value is slightly conservative because the test does not take into account the theoretically higher chance intrusion rate to be expected from unrelated-inconsistent trials). This suggests that while unrelated pairs may have been as capable as related pairs of drawing scene judgments *away* from the actual category of the subsequent scene, as shown by equivalent total error rates, they were significantly less capable of drawing scene judgments *towards* the scene categories associated with their objects. It is worth noting that while each scene in the second block was preceded by the same object pair that preceded it in the first block, any ability of participants to anticipate scenes in the second block based on memory for the objects appearing with them in the first block should only have reduced our ability to detect differences in intrusion rates between conditions by reducing the overall rate of intrusions.

Thus while equivalent total error rates between related- and unrelated-inconsistent pairs was inconsistent with our group-level hypothesis of schema activation, the intrusion imbalance is exactly what one would expect from group-level activation: pairs consisting of objects that were associated with the same scene and therefore as *groups* had strong scene associations were better able to bias decisions towards their associated categories than pairs composed of objects that were associated with different scenes and therefore as groups had comparatively weak associations with any particular scene category. (This is not the only possible interpretation of the differing intrusion rates, as outlined in the Discussion.) However, because the low number of errors in general and of intrusions in particular forced us to pool errors across participants to conduct this analysis, the difference in intrusion rates may have been driven by a small subset of participants. Thus, a second experiment was conducted with a separate group of participants to measure the generality of the intrusion imbalance.

### Experiment 2

This experiment was similar to Experiment 1, but included two changes to increase the number of scene recognition errors committed by participants. First, scene presentation time was reduced to 64 ms, which in a pilot study produced a significant reduction in recognition accuracy relative to the 80 ms presentation time used in Experiment 1. Second, only a total of four scene categories were used, with participants' task taking the form of a four-alternative forced-choice. We reasoned that restricting the scene set in this way would enhance error rates by maintaining a high level of priming for all four scene options. At the same time, the design also reduced the presentation time of object pairs to 32 ms to reduce as much as possible participants' conscious awareness of them, thereby reducing the probability that intrusions might result from conscious weighing of objects' identity. Only three trial types were employed: related-consistent, related-inconsistent, and unrelated-inconsistent.

**Figure 3:**
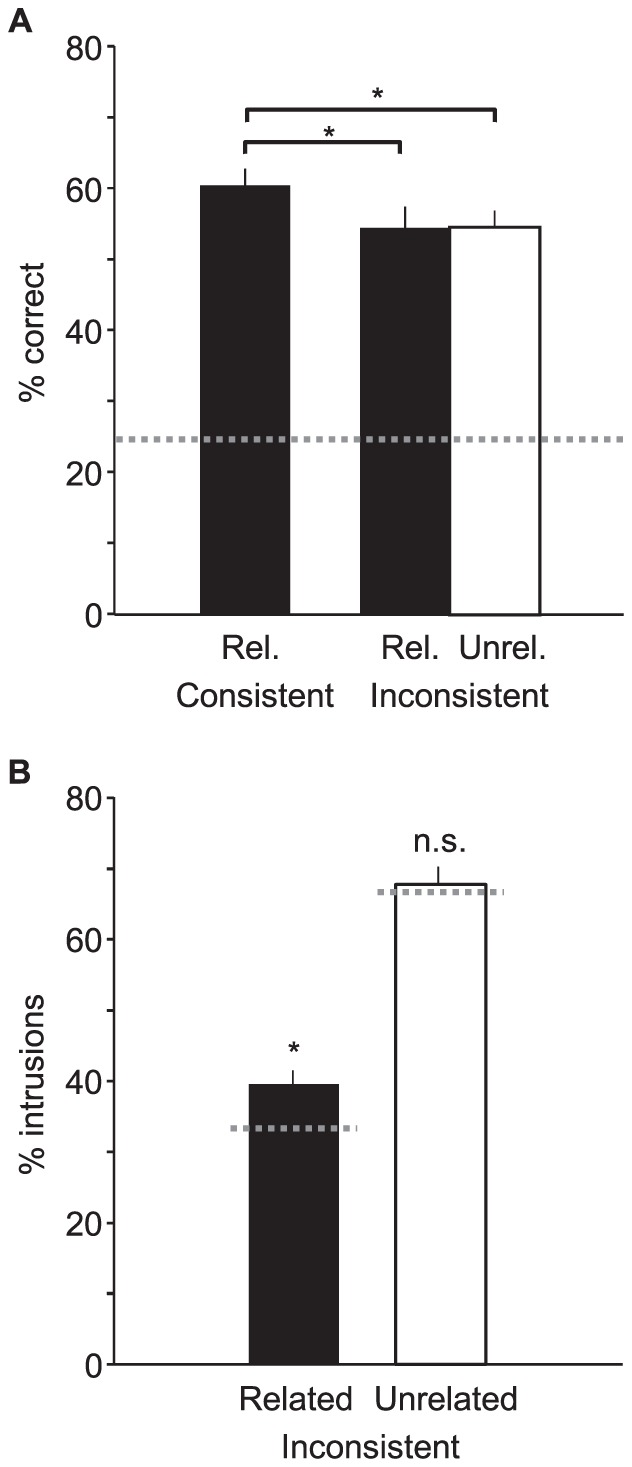
Results of Experiment 2. (a) Recognition accuracy, with dotted line denoting chance performance. (b) Intrusions as a percentage of errors committed on trials with inconsistent objects. Dotted lines denote intrusion rates if all errors resulted from random guessing; significance markers denote significance of observed rates above these chance rates. Error bars are s.e.m. *, p<0.05; n.s. (not significant), p>0.05.

As in Experiment 1, ANOVA showed a significant effect of trial type on scene recognition accuracy (F(2,51) = 6.24, p = 0.0047), and post hoc tests confirmed that accuracy was significantly higher in related-consistent trials than in both related-inconsistent and unrelated-inconsistent trials (p<0.05 for both). Given the near-subliminal presentation times of object pairs, the greater accuracy with consistent objects is not likely to have been an outcome of “guesses” based on conscious awareness of objects' identities. Also consistent with Experiment 1, accuracies in related- and unrelated-inconsistent trials were essentially identical, at 54.3% and 54.6% (Figure 3A).

If categorization errors were outcomes of random guessing, intrusions should have been 33.33% of errors committed on related-inconsistent trials, because 1 of the 3 possible error judgments on each trial matched the scene category associated with the object pair. Similarly, guessing should have resulted in intrusions for 66.67% of errors committed on unrelated-inconsistent trials, for which 2 of the 3 possible error judgments matched the scene category associated with one of the objects. Partly reflecting the shorter object presentation time used in this experiment, but also likely the high steady-state priming of non-intrusion error categories, intrusion rates for both conditions were closer to their respective chance rates than in Experiment 1. However, while intrusion rates in related-inconsistent trials remained significantly above chance (single-sample t-test, t(17) = 3.35, p = 0.002), intrusion rates fell to chance for unrelated-inconsistent trials (t(17) = 0.45, p = 0.33), consistent with the intrusion imbalance observed in Experiment 1 (Figure 3B). Thus as in Experiment 1, while unrelated-inconsistent pairs were as capable as related-inconsistent pairs at inducing errors, their errors were indistinguishable from random guessing, whereas errors committed on related-inconsistent trials showed evidence of object-triggered scene schemata. When directly compared, the excess intrusion rate (i.e., after subtracting the chance rate) observed with related-inconsistent pairs showed a marginally significant increase over the excess intrusion rate observed with unrelated-inconsistent pairs (t(18) = 1.49, p = 0.077).

## Discussion

Although a number of theoretical/computational and experimental studies have shown that scene categories are statistically associated with distinct sets of global properties, and that observers can make category judgments based on these properties [23]–[29], many categories of scenes share global properties to the extent that they can only be practically distinguished from each other on the basis of their object contents. This is particularly clear for many indoor scene categories, but extends to some types of outdoor scenes as well: the distinction, for instance, between a prairie and a farmstead is based upon the presence in the latter of cows, barns, and tractors, etc. Recognizing that not only the identities of objects in scenes but also the relationships among those objects carry information about scene identity, we asked whether simultaneously-viewed objects activate scene schemata as individuals, or as groups. Building upon the previous result that scenes are less accurately recognized when accompanied by objects that are inconsistent with them [8], [9], [17], we predicted that group-level schema generation would be evident as a significant reduction in the negative impact of inconsistent object pairs when the *pairs* were poorly associated with any particular scene category, while the scene associations of *individual* objects within those pairs were held constant.

Against this prediction, in both Experiments 1 and 2 we found that scene recognition accuracy was as negatively impacted by *unrelated*-inconsistent pairs as by *related*-inconsistent pairs. Under the assumption that the magnitude of inconsistent pairs' impact on recognition of subsequently-viewed scenes was proportional to pairs' ability to activate scene schemata, these results suggest that the relationships between objects in pairs did not affect their ability to do so. This result is therefore consistent with item-level schemata activation.

This interpretation is complicated, however, by the pattern of errors we observed. Insofar as the equivalent impact of related- and unrelated-inconsistent pairs on total error counts suggests that they were equally capable of activating scene schemata that competed with actual scenes, they should have produced essentially equal numbers of intrusions – erroneous judgments of scene category matching the scene associated with one or both of each pair's objects. This was not the case: intrusions in both Experiments 1 and 2 were less common in unrelated-inconsistent trials than in related-inconsistent trials. In contrast to the inference drawn from the equivalent total error rates, this intrusion imbalance suggests that unrelated pairs were less capable of activating scene schemata than related pairs. This is consistent with group-level schemata activation.

Thus while a consideration of total error rates suggests that the ability of inconsistent pairs to trigger scene schema was independent of within-pair object relationship, consistent with item-level schemata activation, a comparison between of intrusion rates suggests that schemata triggering did depend upon object relatedness, consistent with group-level schemata activation. Which of these apparently conflicting results is more pertinent? As already mentioned, the support that the equivalence of total error rates offers to item-level schemata generation is predicated on the assumption that excess errors committed with inconsistent pairs (i.e., error counts beyond those observed with consistent objects) reflected activation by those pairs of schemata that competed with subsequent scenes. In contrast, the evidence offered by intrusion rates for group-level activation *requires no such assumption*: inasmuch as intrusions, by definition, involve participants' endorsements of specific scene categories, they provide direct positive evidence of differences in the capacities of related and unrelated pairs to present scene schemata to decision processes. Given this distinction, we believe that relative intrusion rates are more directly relevant to our hypothesis, even if we did not consider them so at the outset of our experiment.

However, while intrusion rates provide strong evidence that unrelated pairs were less capable of presenting object-evoked schemata to decision processes, it is fair to ask whether this difference necessarily reflects group-level schemata *activation*. For instance, could the lower intrusion rates produced by unrelated-inconsistent pairs, instead of resulting from those pairs' relative incapacity to activate unitary schemata at the group level, simply have resulted from decay from short-term memory of multiple item-evoked schemata during the interval between object and scene presentation? To answer this question, let us assume that each unrelated pair activated a pair of schemata that were each as viable at the moment of their activation as the single schema activated by a related pair. We acknowledge that, given some fixed probability that any single activated schema might be forgotten during the object/scene asynchrony, the probability of at least one activated schema being forgotten would necessarily be greater for unrelated pairs than related pairs. By itself, though, this greater loss probability would not be sufficient to explain the lower intrusion rate with unrelated pairs. This is because genuine intrusions (i.e., those not arising from random guesses) only require survival of a single object-activated schema. Thus even if the odds of losing an activated schema were greater for unrelated pairs, any such loss would simply have put those pairs on equal footing with related pairs, which could only have activated single schemata to begin with. The fact that unrelated pairs actually produced fewer intrusions therefore necessarily means that they presented decision processes with numerically fewer active schemata than related pairs did, on average. This is difficult to square with memorial decay, since it would require those pairs activating the greater number of schemata initially (unrelated pairs, which could have activated 2 schemata versus only 1 for related pairs) to result in fewer active schemata at decision time. This would be akin to a participant in a typical short-term memory experiment being less likely to recall at least one item from a large study array than from a small study array. Particularly in light of evidence that short-term memory has a capacity for at least 3 items [31], we therefore consider it more likely that the lower number of object-activated schemata available to decision processes following unrelated pairs reflects their relative incapacity to trigger schemata to begin with.

A related explanation is that multiple item-activated schemata arising from unrelated pairs simply “cancelled” each other. (This cancellation need not have been a time-dependent process, and thus is technically not memory-related.) Our data do not allow us to exclude this possibility. However, our experiments were not designed to offer full proof of group-level processing of objects during scene recognition. Instead, they were designed to assess the viability of the group-level hypothesis by testing the basic prediction that recognition errors should vary with the relationship between items in object ensembles. The intrusion imbalance we observed is consistent with this prediction, although it demands future experiments to confirm its source. Nevertheless, the support our results offer for group-level schemata triggering is consistent with neuroimaging studies demonstrating the existence of specialized neural mechanisms for encoding multiple simultaneously-viewed objects [32]–[35], and that scene categories are encoded by the human brain based on the co-occurrence probabilities of objects within them [15].

Still another potential explanation for lower intrusion rates with unrelated pairs is that the unrelated objects interfered with participants' ability to recognize the objects themselves, thereby naturally degrading the capacity of each to trigger its associated scene schemata. However, rather than conflicting with our group-level hypothesis, we see this as a potential mechanism by which group-level schemata activation might be instantiated: between-object interference ensures that only those object sets that are internally consistent with respect to scene category are eligible to activate schemata. If this were true, though, then why did unrelated pairs produce any above-chance intrusions at all, as they did in Experiment 1? The simplest explanation is that not every unrelated pair resulted in equivalent recognition of both objects. For instance, chance deviations in spatial attention to one object location or the other at the moment of object presentation may have occasionally led to only a single object from an unrelated pair being encoded, thereby negating any potential schema conflict. Any other factors producing an imbalance in the salience of objects in individual trials might similarly have converted the occasional unrelated pair into what was effectively a “related” pair (i.e., one with a single scene association).

Although the intrusion rate difference we observed between related and unrelated pairs that were *inconsistent* with scenes is consistent with group-level schema activation, it is fair to ask whether group-level schema activation has any consequence for scene recognition when objects are *consistent* with their scene, as they often are in the real world. Indeed, we observed that consistent pairs produced no significant benefit to scene recognition over scrambled-object masks, mirroring previous results with single objects [8]. This suggests that, in general, schemata activated by just one or two objects participate little in the recognition of scenes they are consistent with, even if they can significantly disrupt recognition of scenes they are inconsistent with. A direct answer to the question of the relevance of the group- versus item-level distinction to recognition of consistent scenes would require measurement of the effect of within-pair object relationship on intrusions committed against scenes which were consistent with the objects. Unfortunately, it is not logically possible to do so. By the pertinent definition of “related”, a pair that is consistent with a scene is composed of objects that are related to each other. The absence of a fully factorial experiment design in our experiments thus reflects the fact that the pair-scene consistency and within-pair object relatedness are not independent variables. Owing to this, any possible effect of within-pair relatedness can only be observed against the backdrop of inconsistent scenes. We admit, though, that this experimental constraint does not negate the possibility that the group- versus item-level distinction is moot when objects are consistent with scenes, and concede that this may very well be the case. However, while consistency between scenes' object contents and non-object factors (such as global properties) is a common occurrence, it is not uncommon to encounter situations in which objects provide the only cues to a scene's identity. When a visitor explores an unfamiliar house, for example, the high degree of overlap between the global properties of many categories of indoor rooms [30] makes it unlikely that those properties will trigger distinct schemata for any of the categories of scenes likely to be encountered. As such, schemata triggered by objects would presumably be the only ones active, in which case the distinction between group- and item-level activation would certainly rise to relevance, as outlined in the Introduction.

### Sources of scene categorization errors

Regardless of whether the source of the intrusion imbalance was group-level schemata activation or some other factor, how can the imbalance be reconciled with the consistently identical total error rates produced by related and unrelated pairs? Prior studies of the effect of objects on scene recognition have interpreted the negative effect of inconsistent objects on accuracy as an outcome of the activation by those objects of scene schemata which compete with that of the actual scene context [8], [9], [17], [20], and we assumed the validity of this interpretation when we designed our experiments. If this interpretation were correct, however, then the essentially identical total error rates we observed with related- and unrelated-inconsistent pairs should have been accompanied by similarly equivalent intrusion rates. Instead, as we have already emphasized, intrusion rates were higher for related-inconsistent trials.

Inasmuch as intrusion rates provide a more direct measure of schemata activation, our data indicate that the interpretation of object-triggered scene categorization errors as outcomes of object-activated schemata is incorrect. Instead, we propose that excess error rates with inconsistent objects reflected a generic unsigned “conflict signal” that was produced by *any* inconsistency between objects and subsequent scenes. The apparent independence of this signal from the ability of evoking objects to produce intrusions suggests that it reduced scene recognition accuracy by contributing *uncertainty*, not competing schemata, to the interpretation of subsequently-viewed scenes. In this context, intrusions resulted from opportunistic penetration of decision processes by object-activated schemata *after* the conflict signal had already introduced sufficient uncertainty to the interpretation of the actual scene to trigger an error. In sum, we hypothesize that the apparent conflict between total errors and intrusions reflects two independent contributions of objects to scene categorization: a purely *disruptive* influence, mediated by the conflict signal, that was independent of within-pair object relationships and therefore appeared to evaluate objects individually, and a purely *constructive* influence, mediated by object-triggered schemata activation, that shows signs of evaluating objects as groups.

In pointing to separable disruptive and constructive pathways by which objects affect scene recognition, our results are consistent with a recent study showing that the negative effects of inconsistent objects on scene recognition *accuracy* can be explained by those objects' effects on scenes' global properties, such as spatial frequency spectra, rather than activation of semantic networks related to objects' identities [18]. Objects which are inconsistent with a scene were shown to distort the scene's low-level visual statistics, presumably moving them away from those typically associated with the scene's category. Because scene recognition has been shown previously to draw on global properties [23], [26]–[28], [36], these distortions can be expected to reduce scene recognition accuracy without necessarily biasing global properties far enough to generate a scene judgment matching the scene associated with the inconsistent object. Because the source of error is in objects' low-level visual properties, the semantic relationship between a *pair* of inconsistent objects should have no impact on their capacity to reduce recognition accuracy. This is exactly what we observed in our experiments. Thus the “conflict signal” that we have hypothesized to explain our accuracy results may be embodied by inconsistent objects' low-level visual properties.

To our knowledge, ours is the first study to provide data directly testing the assumption that scene recognition errors induced by inconsistent objects result from objects' activation of competing schemata, even if it were not our intent to provide such data when our experiment was designed. Previous studies of the effects of *single* inconsistent objects on scene recognition [8], [17] did not offer a way to test this assumption because they could not independently vary objects' scene consistency and their potential for activating alternative schemata. Similarly, while Davenport [9] explored the effect of object *pairs* on scene recognition, as we did, that study only used pairs composed of related objects, and thus did not permit a comparison of the dependency of error and intrusion rates on between-object relationship while their consistency with scenes they were viewed with was held controlled. In fact, when intrusions were encountered in previous studies [8], [9], they were considered evidence not of direct activation by objects of scene schemata, but of participants' “guessing” scenes' identities based on conscious awareness of and cogitation on objects' identity. Two aspects of our data suggest that this interpretation of intrusions was overly conservative. First, intrusions in our study were committed even when objects were presented essentially subliminally; anecdotal debriefings of participants in Experiment 2 indicated that they had great difficulty naming any objects they had been shown. Second, and more important, the smaller intrusion rate produced by unrelated pairs is difficult to reconcile with any plausible guessing strategy: even if guess-prone participants were confused by unrelated pairs, it seems unlikely that their guesses would have systematically avoided the scene categories associated with the objects. Thus while we cannot rule out that some proportion of intrusions may have resulted from guessing, it seems unlikely that *all* did, and even less so that guessing could have produced the intrusion imbalance we observed.

While our data present a challenge to the idea that objects produce scene categorization errors via activation of competing schemata, a caveat to our interpretation is warranted. Unlike the previous studies we have discussed [8], [9], objects in our study were shown immediately prior to scenes, rather than embedded in them. We employed this asynchronous design because the focus of our study was on how relationships between objects influence activation of scene schemata. If we had embedded objects in scenes, the tangle of relationships between them and scenes' endogenous objects would have made the practical difference between related-inconsistent and unrelated-inconsistent pairs vanishingly small; any effect of the relationship between the embedded objects would have been diluted by the much larger set of uniformly “unrelated” pairwise relationships between the embedded and endogenous objects. The object-scene asynchrony we used was intended to make within-pair relationships more salient by blocking, as much as possible, relationships between object pairs and scenes' endogenous objects. One consequence of this, however, is that the scenes in our experiment arguably did not amount to “contexts” for object pairs in the same way they would have had objected been embedded in them. The impact of this difference is unclear. However, the general agreement between our results and previous work in terms of differences in scene recognition between scene-consistent and inconsistent object pairs suggests that its importance is not great.

In conclusion, we found that the impact of scene-inconsistent object pairs on scene recognition depended on the relationship between objects, although not in the way that we predicted. While pairs that were composed of related and unrelated objects were equally capable of triggering errors in the categorization of subsequent scenes, unrelated object pairs were less capable of biasing erroneous scene judgments towards their associated scene category. The relative inability of unrelated pairs to attract scene judgments towards their associated scene categories is consistent with the hypothesis that scene schemata are triggered, at least in part, by objects evaluated as groups, rather than solely as individual items, though future experiments are necessarily to exclude alternative explanations. Regardless of the source of the intrusion imbalance, however, the fact that related and unrelated pairs produced essentially identical total error counts in spite of evidence for differing capacities to activate scene schemata suggests that excess scene categorization errors produced by scene-object inconsistency are not solely attributable to schema activation by objects. We propose instead that inconsistent objects hinder scene recognition by contributing a purely disruptive “conflict signal”, potentially via a corrupting effect on scenes' global properties, that is independent of the cognitive mechanisms by which they may trigger scene schemata.
